# Aldehyde dehydrogenase-independent bioactivation of nitroglycerin in porcine and bovine blood vessels

**DOI:** 10.1016/j.bcp.2014.12.021

**Published:** 2015-02-15

**Authors:** Regina Neubauer, Gerald Wölkart, Marissa Opelt, Christine Schwarzenegger, Marielies Hofinger, Andrea Neubauer, Alexander Kollau, Kurt Schmidt, Astrid Schrammel, Bernd Mayer

**Affiliations:** Department of Pharmacology and Toxicology, Karl-Franzens-Universität Graz, Austria

**Keywords:** ALDH, aldehyde dehydrogenase, CB25, 1-{[4-(1,3-benzodioxol-5-ylmethyl)-1-piperazinyl]methyl}-1H-indole-2,3-dione, cGMP, 3′,5′-cyclic guanosine monophosphate, DEA/NO, 2,2-diethyl-1-nitroso-oxyhydrazine, DMSO, dimethyl sulfoxide, DPI, diphenyleneiodonium, DTPA, diethylenetriamine pentaacetic acid, DTT, dithiothreitol, EDTA, ethylene diamine tetraacetic acid, GDN, glycerol dinitrate, GTN, glycerol trinitrate (nitroglycerin), NAD, nicotinamide adenine dinucleotide, l-NNA, N^G^-nitro-l-arginine, NO, nitric oxide, ODQ, 1H-[1,2,4]oxadiazolo-[4,3-a]quinoxalin-1-one, sGC, soluble guanylate cyclase., Aldehyde dehydrogenase-2, Denitration, Nitroglycerin, Protein expression, Vascular relaxation

## Abstract

The vascular bioactivation of the antianginal drug nitroglycerin (GTN), yielding 1,2-glycerol dinitrate and nitric oxide or a related activator of soluble guanylate cyclase, is catalyzed by aldehyde dehydrogenase-2 (ALDH2) in rodent and human blood vessels. The essential role of ALDH2 has been confirmed in many studies and is considered as general principle of GTN-induced vasodilation in mammals. However, this view is challenged by an early report showing that diphenyleneiodonium, which we recently characterized as potent ALDH2 inhibitor, has no effect on GTN-induced relaxation of bovine coronary arteries (De La Lande et al., 1996). We investigated this issue and found that inhibition of ALDH2 attenuates GTN-induced coronary vasodilation in isolated perfused rat hearts but has no effect on relaxation to GTN of bovine and porcine coronary arteries. This observation is explained by low levels of ALDH2 protein expression in bovine coronary arteries and several types of porcine blood vessels. ALDH2 mRNA expression and the rates of GTN denitration were similarly low, excluding a significant contribution of ALDH2 to the bioactivation of GTN in these vessels. Attempts to identify the responsible pathway with enzyme inhibitors did not provide conclusive evidence for the involvement of ALDH3A1, cytochrome P450, or GSH-S-transferase. Thus, the present manuscript describes a hitherto unrecognized pathway of GTN bioactivation in bovine and porcine blood vessels. If present in the human vasculature, this pathway might contribute to the therapeutic effects of organic nitrates that are not metabolized by ALDH2.

## Graphical abstract

**Figure:**
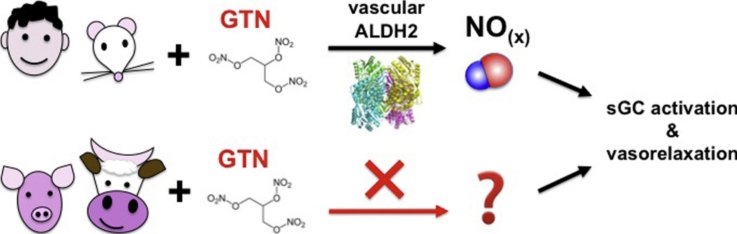

## 1 Introduction

The antianginal drug nitroglycerin (GTN) causes vasodilation through the activation of soluble guanylate cyclase (sGC), resulting in accumulation of 3′,5′-cyclic guanosine monophosphate (cGMP) in vascular smooth muscle. This effect is mediated by enzymatic release of nitric oxide (NO) or a related species, which binds with nanomolar affinity to the prosthetic heme group of sGC. The mechanism underlying bioactivation of GTN to yield NO has remained controversial for several decades. Besides the non-enzymatic reaction of GTN with cysteine [1,2] or ascorbate [3], several enzymes were proposed to catalyze GTN bioactivation, in particular GSH-S-transferase [4,5], cytochrome P450 [6,7], cytochrome P450 reductase [8] and xanthine oxidase, which exhibits nitrite reductase activity at low oxygen tension [9,10]. However, the involvement of xanthine oxidase-catalyzed nitrite reduction was later excluded [11], and neither of the other pathways appears to fully explain GTN vasoactivity [12].

In 2002, Stamler and coworkers proposed aldehyde dehydrogenase-2 (ALDH2) as the key enzyme that catalyzes GTN bioactivation [13], and meanwhile there is general agreement that this pathway is essentially involved in GTN-induced relaxation in rodent and human blood vessels [14]. The evidence is based on inhibition of GTN-induced relaxation by ALDH2 inhibitors [13] and the absence of the high affinity pathway of GTN vasodilation in ALDH2-deficient mice [15]. The main route of ALDH2-catalyzed denitration of GTN yields 1,2-glycerol dinitrate (1,2-GDN) and inorganic nitrite, but our data obtained with several ALDH2 mutants suggest that GTN bioactivity is mediated by a minor pathway resulting in the direct formation of NO [16].

The involvement of ALDH2 in GTN-induced relaxation has been demonstrated for rabbit [13], rat [17–19], mouse [15,20], guinea pig [21], and human [22,23] blood vessels, suggesting a general principle of vascular GTN bioactivation in mammals. However, in an early study Horowitz and coworkers showed that diphenyleneiodonium (DPI), which we recently characterized as potent ALDH2 inhibitor [24], does not affect relaxation to GTN of bovine coronary arteries [25]. In the present study we confirm this observation, extend it to porcine coronary arteries, and show that ALDH2 expression is very low in bovine coronary arteries and several types of porcine vessels. The results point to an ALDH2-independent pathway of GTN bioactivation that may be relevant for the pharmacology of organic nitrates, in particular the isosorbide nitrates, which are not metabolized by ALDH2.

## 2 Materials and methods

### 2.1 Materials

Human ALDH2 was expressed in *Escherichia coli* BL21 (DE3) and purified as described previously [26,27]. Ethylenediamine tetraacetic acid-(EDTA)-free Complete™ Protease Inhibitor Cocktail Tablets were from Roche Diagnostics GmbH (Vienna, Austria). [2-^14^C]GTN (50 mCi/mmol) was from American Radiolabeled Compounds, purchased through Hartmann Analytic GmbH (Braunschweig, Germany). Nitro POHL^®^ ampoules (G. Pohl-Boskamp GmbH & Co., Hohenlockstedt, Germany), containing 4.4 mM GTN in 250 mM glucose, were obtained from a local pharmacy and diluted with distilled water. Unlabelled organic nitrates used as standards in radio thin layer chromatography (GTN, 1,2-GDN and 1,3-GDN) were purchased from LGC Standards (Wesel, Germany). 2,2-Diethyl-1-nitroso-oxyhydrazine (DEA/NO) and 1H-[1,2,4]oxadiazolo-[4,3-a]quinoxalin-1-one (ODQ) were from Enzo Life Sciences (Lausen, Switzerland) purchased through Eubio (Vienna, Austria). DEA/NO was dissolved and diluted in 10 mM NaOH. The selective ALDH3A1 inhibitor 1-{[4-(1,3-benzodioxol-5-ylmethyl)-1-piperazinyl]methyl}-1H-indole-2,3-dione (CB25) [28] was obtained from ChemBridge Corporation (San Diego, CA, USA). All other chemicals were from Sigma–Aldrich (Vienna, Austria), including 9,11-dideoxy-11α,9α-epoxymethanoprostaglandin F2α (U-46619), DPI, N^G^-Nitro-l-arginine (l-NNA) and chloral hydrate. Stock solutions of ODQ (100 mM), DPI (10 mM) and U-46619 (0.1 mM) were prepared in dimethyl sulfoxide or ethanol and further diluted in buffer. Final concentration of organic solvents did not exceed 0.1%.

### 2.2 Animals and tissues

Sprague-Dawley rats (obtained from Charles River, Sulzfeld, Germany) of either sex were housed at the local animal facility in approved cages and kept on a regular 12-hour dark/light cycle. They were fed standard chow (Altromin 3023; obtained from Königshofer Futtermittel (Ebergassing, Austria)) and received water *ad libitum*. Animals were euthanized in a box that was gradually filled with CO_2_ until no more vital signs (cessation of respiration and circulation) were noticed. Subsequently, the thorax of the animals was opened. The thoracic aorta (and for some experiments heart and liver) were removed and placed in chilled buffer.

Porcine and bovine hearts were obtained from a local abattoir and immediately transported to the laboratory. The right coronary artery was carefully explanted and immediately used for assessment of vessel function.

### 2.3 Ring experiments (organ bath)

For isometric tension measurements, isolated blood vessels were cut into rings of ∼3 mm length, and the rings suspended in 5-ml organ baths, containing oxygenated Krebs–Henseleit buffer (concentrations in mM: NaCl 118.4, NaHCO_3_ 25, KCl 4.7, KH_2_PO_4_ 1.2, CaCl_2_ 2.5, MgCl_2_ 1.2, d-glucose 10.1; pH 7.4) as previously described in detail [24]. After equilibration for 60 min at the optimal resting tension (rat aorta: 1 g; porcine coronary artery: 2 g; bovine coronary artery: 4 g), maximal contractile activity was determined with a depolarizing solution containing 100 mM K^+^. Rings that did not elicit adequate and stable contraction to high K^+^ were considered as being damaged and omitted from the study. After washout, tissues were precontracted with the thromboxane mimetic U-46619. Addition of 50 nM U-46619 contracted rat aortas to ∼90% of contraction obtained with 100 mM K^+^, while 50 nM U-46619 contracted porcine and bovine coronary arteries to ∼60% of maximal contraction. If indicated, chloral hydrate (1 mM), DPI (0.3 μM), l-NNA (1 mM) or ODQ (100 μM) were added simultaneously with U-46619 to the preparation. After a stable tone had been reached (∼20 min), cumulative concentration–response curves were established in separate rings with GTN (0.1 nM–300 μM) or DEA/NO (1 nM–10 μM). The contractile force corresponding to each agonist concentration was recorded and expressed as percent of precontraction. For comparison of agonist potency in aortas and coronary arteries, concentration–response curves to GTN and DEA/NO were additionally established with rat aortic rings precontracted with 20 nM instead of 50 nM U-46619 (yielding ∼50% of maximal contraction; see Legend to Fig. 2).

### 2.4 Measurement of coronary flow in perfused isolated rat hearts

Hearts from euthanized animals were mounted on a heart perfusion apparatus (Hugo Sachs Elektronik/Harvard Instruments, March-Hugstetten, Germany) and retrogradely perfused at 37 °C with oxygenated Krebs–Henseleit buffer at 80 mm Hg (constant pressure perfusion) as previously described [21]. The following cardiac parameters were monitored in unpaced hearts: coronary flow (as index of coronary arterial function) with a transonic flow probe, left-ventricular developed pressure *via* a fluid-filled balloon that was inserted into the left ventricle and connected to a pressure transducer, and heart rate, derived electronically from the pressure signal.

After equilibration for 30 min (baseline), coronary relaxation was induced with GTN given as bolus injections through a sideline in non-cumulative manner, resulting in final concentrations of ∼1 nM to 100 μM (5 min per dose). After the last dose, GTN was washed out for 30 min, and baseline was re-established. Thereafter, a concentration–response curve to DEA/NO (1 nM–10 μM) was established (total duration of the experiment 120 min). To test for the involvement of ALDH2, experiments were performed in the absence and presence of 0.1 μM DPI, added to the perfusion buffer.

### 2.5 Immunoblotting

Freshly isolated aortas or coronary arteries were cleaned, weighed and pre-digested with collagenase (1.5 mg/ml) in 10 mM Tris-buffer, pH 7.4, containing 250 mM sucrose, 3 mM CaCl_2_, 100 U/ml penicillin, 0.1 mg/ml streptomycin, and 1.25 μg/ml amphotericin B for 30 min at 37 °C. After incubation, tissues were washed in phosphate-buffered saline and homogenized with a Potter-Elvehjem glass or teflon homogenizer in 5–10 fold volumes of 10 mM Tris-buffer, pH 7.4, containing 125 mM potassium chloride, 5 mM ethylene glycol tetraacetic acid, 2 mM MgCl_2_ and Complete™ Protease Inhibitor Cocktail (Roche Diagnostics GmbH, Vienna, Austria). Osmolarity was adjusted to ∼290 mosmol/l with NaCl. Non-fibrous liver tissues were homogenized directly after isolation. Homogenates were centrifuged at 510 × *g* and 4 °C for 5 min to remove tissue debris and then centrifuged at 20,800 × *g* and 4 °C for 10 min to separate membranes and organelles, including mitochondria. Supernatants were regarded as cytosolic fractions, the obtained pellets were washed in 0.4 ml buffer, centrifuged again at 20,800 × *g* and 4 °C for 10 min. The resulting pellets are designated as mitochondrial fractions throughout the paper. For preparation of total homogenates, tissues were weighed, homogenized in a 10-fold volume of buffer and centrifuged at 510 × *g* and 4 °C for 5 min Protein concentration was determined with the Pierce BCA™ Protein Assay Kit (Fisher Scientific Austria GmbH, Vienna, Austria). Denatured samples (10–40 μg of protein) were separated by sodium dodecyl sulfate polyacrylamide gel electrophoresis on 12% gels and transferred onto nitrocellulose membranes. After blocking with 5% nonfat dry milk in phosphate-buffered saline, containing 0.05% Tween-20 (v/v), for 1 h membranes were incubated overnight at 4 °C with primary antibodies against human ALDH2 (1:20,000; polyclonal, kindly provided by Dr. Henry Weiner), β-actin (1:200,000; Sigma), citrate synthetase (CS, 1:1000; Abcam) and glyceraldehyde-3-phosphate dehydrogenase (GAPDH; 1:50,000; Sigma). After incubation of membranes with horseradish peroxidase-conjugated anti-rabbit or anti-mouse IgG (1:5000), immunoreactive bands were visualized by chemiluminescence using ECL Prime Western Blot Detection Reagent (GE Healthcare, purchased *via* VWR, Vienna, Austria) and quantified densitometrically using the E.A.S.Y. Win 32 software (Herolab, Vienna, Austria).

### 2.6 Determination of ALDH mRNA expression

Total RNA was isolated from homogenized tissues (rat aorta, porcine and bovine coronary arteries) using the GenElute™Mammalian Total RNA Miniprep Kit (Sigma) including DNAse treatment of samples. cDNA was synthesized using the High Capacity DNA Reverse Transcription Kit (Applied Biosystems, Vienna, Austria). Primers for mRNA expression analysis were designed on the basis of published rat, porcine and bovine nucleic acid sequences of GenBank (NCBI) using the Primer-BLAST software (Table 1). Amplification efficiency of the primers was determined by qPCR analysis using serial dilutions of the cDNA template. Efficiency was calculated from the slope of the curve using the following equation: *E* = 10^(−1/slope)^ (Table 1). Real-time PCR analysis was performed with ∼10 ng cDNA and primer concentrations of 100 nM using Power SYBR^®^ Green PCR Master Mix (Applied Biosystems, Vienna, Austria). Reactions were carried out on a 7300 Real-Time PCR System (Applied Biosystems, Vienna, Austria). Cycling conditions were as follows: 2 min at 50 °C, 10 min at 95 °C, 50 cycles of 15 s at 95 °C, and for 1 min at 60 °C. Melting curve analysis consisted of the following steps: 15 s at 95 °C, 30 s at 60 °C, and 15 s at 95 °C. Data were analyzed and calculated according to Pfaffl [29]. Data were calculated relative to rat aortic ALDH2 mRNA expression after normalization to cyclophilin A.

### 2.7 GTN denitration catalyzed by intact blood vessels

The rates of GTN denitration catalyzed by rat aortas as well as porcine and bovine coronary arteries were determined as formation of 1,2- and 1,3-GDN in the absence and presence of the ALDH2 inhibitor chloral hydrate. Freshly removed arteries were cleaned, weighed and cut into four pieces each, which were equilibrated at 37 °C for 15 min in organ baths containing 5 ml of oxygenated phosphate buffer (50 mM K_2_HPO_4_, 80 mM NaCl, 3 mM MgCl_2_, 2 mM EDTA and 1 mM NAD^+^, pH 7.4). Afterwards, pieces were transferred into a final volume of 0.2 ml of the same buffer, diced with scissors, and incubated for 10 min at 37 °C in the presence of 2 μM ^14^C-labeled GTN and 2 mM dithiothreitol (DTT). Reactions were terminated by flash freezing the samples twice in liquid nitrogen [30]. GTN and denitrated metabolites were extracted twice with 1 ml diethyl ether, separated by thin layer chromatography, and quantified by liquid scintillation counting as described previously [18]. Blank values were determined in the absence of tissue under identical conditions and subtracted.

### 2.8 GTN denitration catalyzed by subcellular fractions of rat liver, heart, and aorta

Freshly removed tissues were cleaned, weighed and homogenized gently with a teflon potter in 10 mM Tris-buffer, pH 7.4, containing 125 mM KCl, 5 mM ethylene glycol tetraacetic acid, 2 mM MgCl_2_, and Complete™ Protease Inhibitor Cocktail. If necessary, osmolarity was adjusted to ∼290 mosmol/l with NaCl. Homogenates were centrifuged at 510 × *g* and 4 °C for 5 min to remove tissue debris and nuclei, followed by centrifugation at 20,800 × *g* and 4 °C for 10 min to pellet mitochondria. Supernatants were regarded as cytosolic fractions, pellets were washed with 0.4 ml buffer, centrifuged again at 20,800 × *g* and 4 °C for 10 min, resuspended in equal volumes of buffer as cytosols and used as mitochondrial fractions. The rates of GTN denitration were determined as formation of 1,2- and 1,3-GDN as described previously [18]. Subcellular fractions obtained from 10 to 20 mg of tissue were incubated with 2 μM ^14^C-labeled GTN at 37 °C for 10 min in a final volume of 0.2 ml of 50 mM potassium phosphate buffer, pH 7.4, containing 3 mM MgCl_2_, 2 mM EDTA, 1 mM NAD^+^, and 2 mM DTT in the absence and presence of the ALDH2 inhibitor chloral hydrate. Samples were analyzed for ^14^C-labeled 1,2-GDN as described above.

### 2.9 Statistical analysis

Data are presented as mean values ± SEM of *n* experiments. Concentration–response curves established with different ring segments from a single animal were averaged and counted as individual experiment (*n* = 1). Individual concentration–response curves were fitted to a Hill-type model giving estimates of agonist potency (EC_50_) and efficacy (*E*_max_). Analysis of variance (ANOVA) with *post hoc* Bonferroni–Dunn test was used for comparison between groups using StatView^®^ (Version 5.0). Significance was assumed at *p* < 0.05.

## 3 Results

### 3.1 GTN causes ALDH2-independent relaxation of porcine and bovine coronaries

**Fig. 1:**
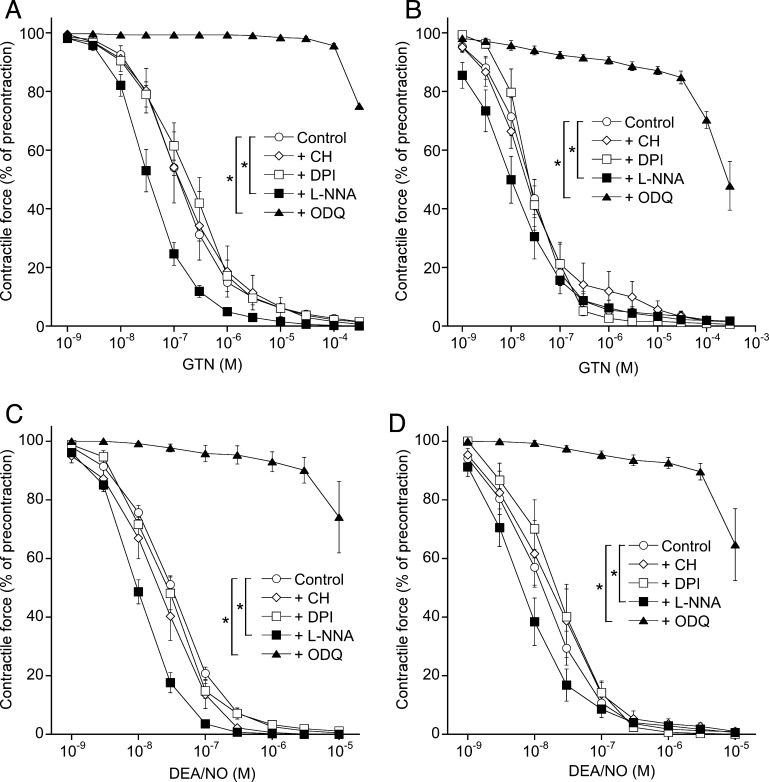
Effects of chloral hydrate, DPI, l-NNA and ODQ on relaxation of porcine and bovine coronary arteries to GTN and DEA/NO. Rings from porcine (A, C) and bovine (B, D) coronary arteries were precontracted with U-46619 (50 nM) in the absence or presence of chloral hydrate (CH; 1 mM), DPI (0.3 μM), l-NNA (1 mM), and ODQ (0.1 mM). Cumulative concentration–response curves to GTN (A, B) or DEA/NO (C, D) were established. Data obtained with two different ring segments from the same vessel were averaged and counted as individual experiment (*n* = 1). The results shown are mean values ± SEM of 3–5 (inhibitors) or 12–35 (controls) experiments (*p < 0.05).

Fig. 1 shows the relaxation of porcine (A) and bovine (B) coronary arteries to GTN with EC_50_ values of 146 ± 14 and 32 ± 7 nM, respectively. Surprisingly, relaxation was not affected by the non-selective ALDH inhibitor chloral hydrate or DPI, which we have recently described as potent ALDH2 inhibitor [24]. Relaxation to GTN of porcine coronary arteries which had been precontracted with 30 mM KCl instead of U-46619 was also insensitive to chloral hydrate (data not shown). Since these results suggested that ALDH2 does not contribute to GTN bioactivation in these vessels, we tested for the potential involvement of endogenous NO [31] or a pathway unrelated to sGC activation. However, the NO synthase inhibitor l-NNA did not antagonize but even slightly potentiated the effect of GTN (EC_50_ = 40 ± 8 and 14 ± 4 nM in porcine and bovine vessels, respectively). Complete inhibition of relaxation by the sGC inhibitor ODQ [32] strongly indicates that activation of sGC by GTN-derived NO or a related species is essentially involved. The direct NO donor DEA/NO caused relaxation of porcine and bovine coronary arteries with EC_50_ values of 38 ± 4 and 18 ± 4 nM, respectively (panels C and D). Again, chloral hydrate and DPI had no effect, relaxation was slightly potentiated by l-NNA and blocked by ODQ.

**Fig. 2:**
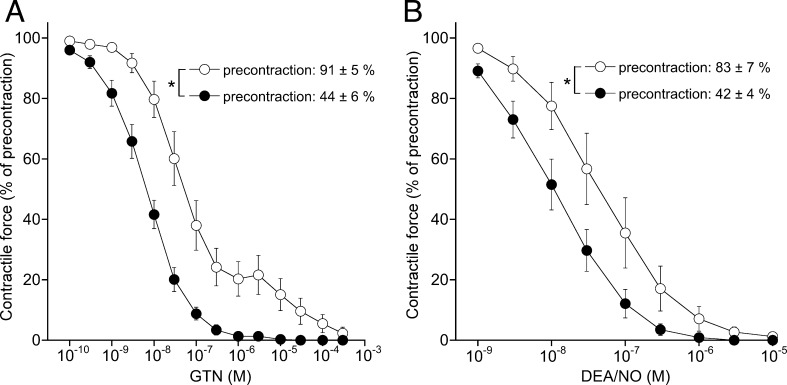
Effects of precontraction levels on relaxation of rat aortic rings to GTN and DEA/NO. Concentration response to GTN (A) and DEA/NO (B) of rat aortic rings precontracted with 50 nM (open symbols) or 20 nM (filled symbols) U-46619, resulting in precontraction levels of 91 ± 5% and 44 ± 6% (A) and 83 ± 7% and 42 ± 4% (B), respectively. The corresponding EC_50_ values were 81.2 ± 38 and 8.3 ± 2.1 nM (A) and 79.4 ± 38 and 15.4 ± 5.4 nM (B). Data are mean values ± SEM of five experiments (*p < 0.05).

As compared to the bulk of data obtained previously by us and others with rat and mouse aortas, the present data apparently point to significantly higher GTN and NO sensitivity of porcine and, in particular, bovine coronary arteries. Moreover, the typical biphasic concentration–response to GTN observed with the rodent vessels was absent. This difference is probably a consequence of the lower level of precontraction applied in the experiments with coronary arteries. As illustrated in Fig. 2, both GTN (A) and DEA/NO (B) dilated rat aortic rings with 5- to 10-fold higher potency when the precontraction level was reduced from 80 to 90% (as typically applied) to about 40%. Note that potentiation of the GTN effect led to virtually complete relaxation through the high affinity pathway.

Regardless of precontraction levels, the potency of GTN relative to that of DEA/NO varied considerably within species. In rat aorta, the high affinity pathway of GTN-induced relaxation exhibited an EC_50_ of 86 ± 5 nM as compared to 183 ± 22 nM determined for DEA/NO (data not shown). Thus, GTN was about 2-fold more potent than DEA/NO in rat aorta, presumably due to site-specific intracellular release of GTN-derived NO or a related bioactive species. In contrast, GTN was about 4- and 2-fold less potent than DEA/NO in porcine and bovine coronaries (ratios of EC_50_ values = 0.26 and 0.56, respectively). Thus, the ALDH2-independent GTN bioactivation in porcine and bovine blood vessels appears to take place with around 5-fold lower GTN potency than ALDH2-mediated relaxation.

**Fig. 3:**
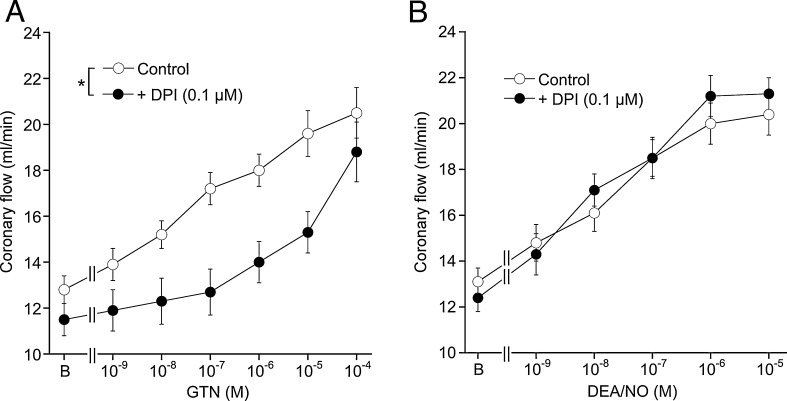
Effects of ALDH2 inhibition on coronary vasodilation induced by GTN and DEA/NO in isolated perfused rat hearts. Hearts were perfused at a constant pressure of 80 mm Hg in the absence or presence of 0.1 μM DPI. Increase of coronay flow, reflecting microvascular vasodilation, caused by GTN (A) or DEA/NO (B) was recorded. Data are mean values ± SEM of six hearts per group. *p < 0.05 for 10^−9^ to 10^−5^ M GTN (panel A).

In view of the overwhelming evidence for an essential role of ALDH2 for GTN bioactivation in rodent and human blood vessels, the ALDH2-independent effects of the nitrate in porcine and bovine coronary arteries were unexpected. To find out whether this is due to a species difference or reflects a specific feature of coronary arteries, we used isolated rat hearts perfused in the Langendorff mode to test for the involvement of ALDH2 in coronary relaxation to GTN. Fig. 3 shows that the increase in coronary flow caused by GTN was markedly inhibited by the ALDH2 inhibitor DPI (panel A), whereas relaxation to DEA/NO was not affected (panel B). Thus, the striking difference between rodent and porcine/bovine blood vessels is independent of the vascular bed and apparently due to a species difference in the molecular mechanism underlying vascular GTN bioactivation.

### 3.2 ALDH2 protein and mRNA expression in porcine and bovine blood vessels

**Fig. 4:**
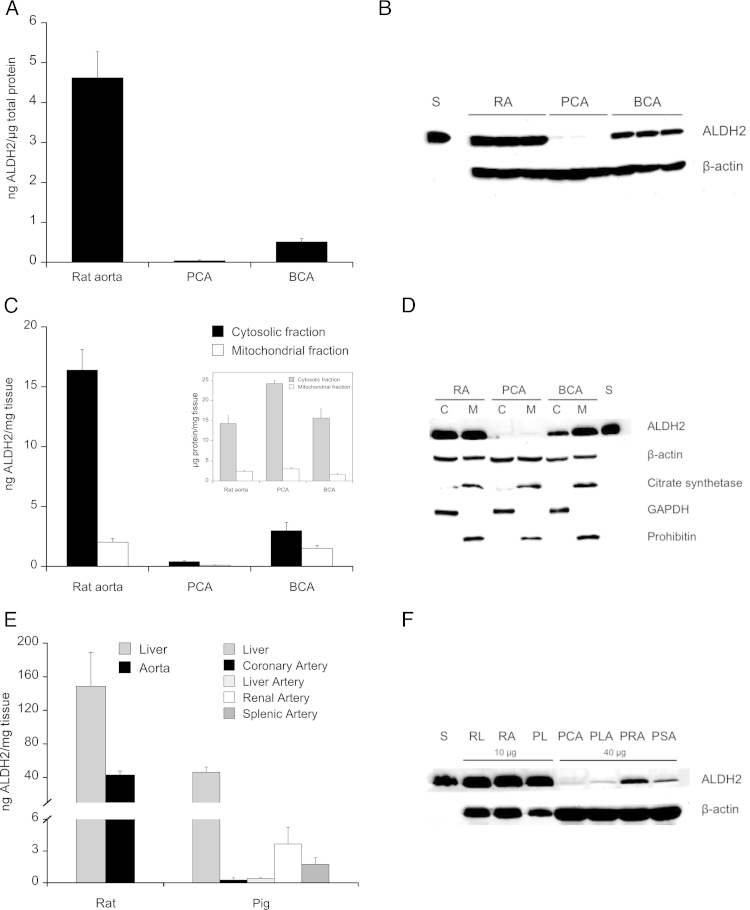
ALDH2 expression in liver and blood vessels of rats, pigs, and cows. (A) ALDH2 expression in rat aorta (RA), porcine coronary arteries (PCA), and bovine coronary arteries (BCA) (*n* = 3). (B) Representative Western blot of total homogenates (30 μg of protein), showing ALDH2 (54 kDa) and β-actin (43 kDa). (C) Subcellular distribution of ALDH2 in RA, PCA, and BCA (*n* = 4–6). (D) Representative Western blot of mitochondrial (M) and cytosolic (C) fractions (25 μg of protein each), showing ALDH2 (54 kDa) and β-actin (43 kDa). Citrate synthetase (52 kDa) and prohibitin (30 kDa) as well as GAPDH (37 kDa) are shown as mitochondrial and cytosolic marker proteins, respectively. Note the unequal protein yields obtained in the course of fractionation (shown in the inset to panel C), precluding estimation of protein distribution by visual inspection of band intensities. (E) Expression of ALDH2 in rat liver (RL), rat aorta (RA), and porcine liver (PL), as well as the following porcine blood vessels: coronary artery (PCA), liver artery (PLA), renal artery (PRA), and splenic artery (PSA) (*n* = 4). (F) Representative Western blots of total homogenates showing ALDH2 (54 kDa) and β-actin (43 kDa). Purified human ALDH2 (25 ng) was used as standard (S) for protein quantification. Summary data are expressed as ng ALDH2 per μg total protein or mg wet weight and represent mean values ± SEM of the number of experiments indicated in panel descriptions.

To shed light on this issue, we studied ALDH2 expression in these blood vessels by quantitative immunoblotting using human ALDH2 as a standard protein. As shown in Fig. 4A, homogenates of rat aorta contained 4.6 ± 0.65 ng of ALDH2 per μg of total protein. Expression of ALDH2 was much lower in porcine and bovine coronary arteries (0.04 ± 0.02 and 0.51 ± 0.08 ng/μg of total protein). A representative blot is shown in Fig. 4B. The subcellular distribution of ALDH2 is shown in Fig. 4C (summary data) and Fig. 4D (representative blot). Rat aorta contained 16.4 ± 1.73 and 2.0 ± 0.31 ng of ALDH2 per mg wet weight in cytosolic and mitochondrial fractions, respectively, confirming the predominant cytosolic localization of ALDH2 in rodent blood vessels observed previously [17,33]. Expression levels were considerably lower in the bovine vessels (3.0 ± 0.69 and 1.5 ± 0.25 ng/mg in cytosolic and mitochondrial fractions, respectively). In porcine coronary arteries ALDH2 was hardly detectable (<0.5 ng/mg). Note that pronounced differences in the recovery of cytosolic and mitochondrial protein (shown in the inset to Fig. 4C) may mislead judgment of band intensities in the blot (Fig. 4D), which was loaded with identical amounts of cytosolic and mitochondrial protein.

**Fig. 5:**
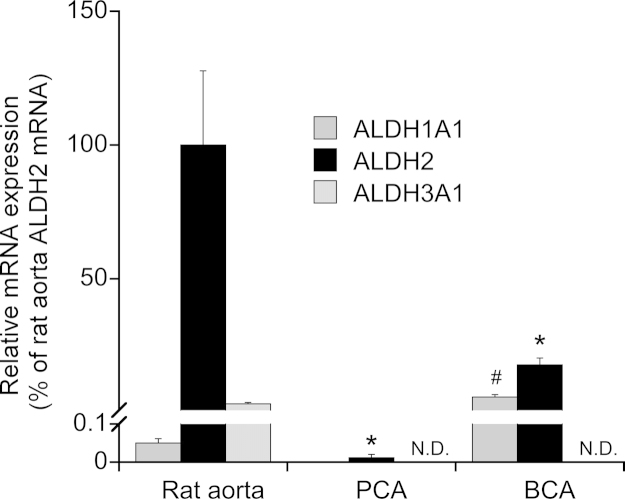
Relative mRNA expression of ALDH isoforms in rat aorta, and porcine and bovine coronary arteries. Total RNA was isolated from rat aortas (*n* = 5), and bovine and porcine coronary arteries (*n* = 5–6). mRNA levels were determined by SYBR Green-based qPCR, and expression levels are calculated relative to the ALDH2 mRNA levels of rat aortas after normalization to cyclophilin A. Data are mean values ± SEM of 5–6 experiments.; n.d., not detectable (<0.0001%). ALDH1A1 mRNA was not determined in PCA. *p < 0.05 compared with ALDH2 in rat aorta; ^#^*p* < 0.05 compared with ALDH1A1 in rat aorta.

To see whether low ALDH2 expression is a peculiarity of coronary arteries, we measured the protein levels in several types of porcine blood vessels and compared the data with the ALDH2 expression levels in rat liver, rat aorta, and porcine liver. As shown in Fig. 4E, the highest expression levels were found in liver, containing about 150 (rat) and 40 (pig) ng/mg wet weight. Rat aorta expressed about 40 ng of ALDH2 per mg tissue, whereas expression levels were below 5 ng/mg in all porcine arteries studied (coronary, liver, renal, and splenic artery). The data on protein levels agreed well with ALDH2 mRNA expression (Fig. 5), which was hardly detectable in porcine coronary arteries (∼0.01% of rat aorta) and about 18% of rat aortic levels in bovine coronary arteries. Expression levels of ALDH1A1 and ALDH3A1, respectively, were about 0.05 and 3% of the ALDH2 levels in rat aorta, and not or hardly detectable in porcine and bovine coronary arteries.

### 3.3 Low rates of GTN denitration in porcine and bovine coronaries

**Fig. 6:**
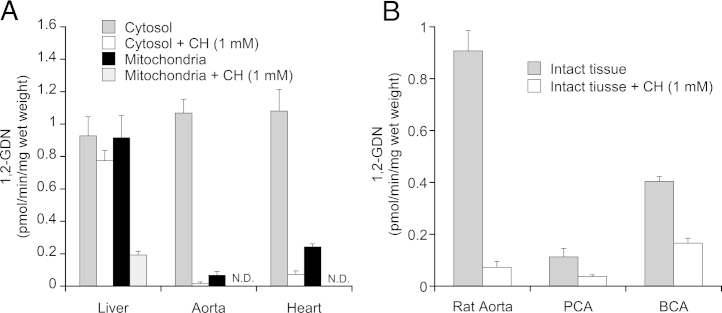
GTN denitration activity of vascular and non-vascular tissues. GTN denitration was measured by incubation of tissues in the absence and presence of chloral hydrate (CH; 1 mM) with 2 μM [^14^C]GTN at 37 °C for 10 min in 0.2 ml of 50 mM potassium phosphate buffer, pH 7.4, containing 3 mM MgCl_2_, 2 mM EDTA, 1 mM NAD^+^, and 2 mM DTT. 1,2-GDN was extracted and analyzed by radio thin layer chromatography as described in Section 2. A, Rates of 1,2-GDN formation by cytosolic and mitochondrial fractions obtained from 10 to 20 mg of rat liver, aorta, and heart. Data are mean values ± SEM of 4–5 independent experiments. B, Rates of 1,2-GDN formation by rat aorta (RA), porcine coronary arteries (PCA), and bovine coronary arteries (BCA) in the absence and presence of 1 mM CH. Data are mean values ± SEM of three independent experiments.

The rates of GTN denitration were assayed as formation of 1,2-GDN and 1,3-GDN by blood vessel homogenates or subcellular fractions. For comparison and validation of the method, we studied GTN denitration by rat liver, aorta and heart in the absence and presence of chloral hydrate. As shown in Fig. 6A, similar denitration activities were measured in cytosolic and mitochondrial fractions of rat liver, but only mitochondrial denitration was sensitive to chloral hydrate, confirming that mitochondrial ALDH2 catalyzes GTN denitration in liver. In contrast, ALDH2-catalyzed GTN denitration activity was almost exclusively cytosolic in rat aorta and mainly cytosolic in rat heart. As shown in Fig. 6B, the rates of GTN denitration measured in pieces of equilibrated intact pig and bovine coronary arteries were markedly lower than in rat aortic rings. These results agree well with the low levels of ALDH2 protein and mRNA expression in these blood vessels. The rates of 1,3-GDN formation were very low under all experimental conditions and are not shown for the sake of clarity.

### 3.4 Attempts to identify the pathway of GTN bioactivation

In search for possible enzymatic pathways of GTN bioactivation on porcine and bovine coronary arteries we considered the involvement of ALDH3A1 [34], cytochrome P450, GSH-S-transferase, γ-glutamylcysteine synthetase, and xanthine oxidase.

The selective ALDH3A1 inhibitor CB25 [28] inhibited aldehyde dehydrogenase activity in homogenates of rat eyes, which contain large amounts of ALDH3A1 [35], with an IC_50_ of 5.7 ± 2.2 μM but had no effect on GTN- or DEA/NO-induced relaxation of rat aorta or coronary arteries from pigs and cows (data not shown).

The cytochrome P450 inhibitor 7-ethoxyresorufin (2 μM) had no effect on relaxation induced by either GTN or DEA/NO. SKF-525A (proadifen, 0.05–0.5 mM) relaxed the vessels in the absence of donors, precluding further testing. Ketoconazole (10 μM) caused a significant 2-fold rightward shift of the GTN response without affecting relaxation to DEA/NO. Although this finding suggests a minor contribution of a cytochrome P450, this pathway is certainly not the major route of ALDH2-independent GTN bioactivation in porcine coronary arteries.

The GSH-S-transferase inhibitor bromosulfophthalein (1 mM) blocked contraction to U-46619 and inhibited relaxation of K^+^-contracted vessels to both GTN and DEA/NO. Ethacrynic acid (0.1 mM) had no effect on relaxation of either donor, and 2,4-dinitrochlorobenzene (0.4 mM) caused unstable contractions, precluding further studies. Finally, inhibition of γ-glutamylcysteine synthetase and xanthine oxidase with buthionine sulfoximine (10 μM) and allopurinol (0.1 mM), respectively, had no effect on relaxation to GTN or DEA/NO (data not shown).

Finally, we considered a possible direct action of GTN on large conductance Ca^2+^-activated K^+^ channels using the established inhibitor iberiotoxin [36]. The drug inhibited relaxation to GTN and DEA/NO in a similar manner (data not shown), confirming that large conductance Ca^2+^-activated K^+^ channels contribute to NO-triggered relaxation [37]. However, the data provide no evidence for a specific involvement of these channels in GTN-induced vasodilation.

## 4 Discussion

Numerous studies have confirmed the essential role of ALDH2 in vascular GTN bioactivation, originally proposed by Stamler and coworkers in 2002 [13]. Besides inhibition of GTN-induced relaxation by various ALDH2 inhibitors, including non-selective compounds such as chloral hydrate and cyanamide [13], as well as the ALDH2-selective inhibitors daidzin [3,38], and DPI [24], loss of the high affinity pathway of GTN-induced vasodilation upon deletion of the ALDH2 gene in mice [15] provided conclusive evidence for the involvement of ALDH2 in GTN bioactivation. Since similar results were obtained with blood vessels from several rodent species (mouse, rat, guinea pig) as well as human arteries [22] and veins [23], the ALDH2 reaction is widely considered as a general principle of GTN bioactivation in mammalian vascular tissue.

However, in the 1990s Horowitz and coworkers reported that DPI, which we recently identified as potent ALDH2 inhibitor, had no effect on GTN-induced relaxation of bovine coronary arteries [25]. In view of current knowledge this observation is surprising and hard to reconcile with the ALDH2 hypothesis of GTN bioactivation. The present study explains this astounding observation as a consequence of low ALDH2 expression and GTN denitration activity. The protein was hardly detectable in porcine coronary arteries, while significant amounts were found in the bovine vessels (albeit still much lower than in rat aorta). A similar pattern was observed for the rates of denitration, which were high in rat aorta and very low in porcine coronaries, while bovine coronaries exhibited about 50% of the activity measured with rat aorta. Based on this observation one might expect a significant contribution of ALDH2 to relaxation of bovine vessels, which was not observed. However, the difference is more pronounced after subtraction of ALDH2-independent denitration, yielding rates of 0.84 and 0.23 pmol min^−1^ mg^−1^ for rat aorta and bovine coronaries, respectively. Moreover, there was a significant difference in the subcellular distribution of ALDH2 in the two types of blood vessels. While about 90% of the protein was cytosolic in rat aorta, equal amounts of ALDH2 were found in cytosolic and mitochondrial fractions of bovine coronary arteries (cf. Fig. 4C). Since cytosolic expression of ALDH2 appears to be essential for vascular GTN bioactivation [33], significant mitochondrial localization of the protein may further reduce the fraction of enzyme available for GTN bioactivation in the bovine vessels. We cannot exclude, however, a minor contribution of ALDH2 to relaxation that was not detectable in the organ bath experiments.

Virtually complete inhibition of GTN-induced relaxation by ODQ indicates that vasodilation was caused by activation of sGC. Since GTN does not activate sGC directly, the effect apparently involves an enzymatic or non-enzymatic reaction yielding a NO-like bioactive species together with denitrated metabolites. At a first glance, the low denitration rates we observed with porcine and coronary arteries seem to be inconsistent with this assumption. However, we have previously shown that ALDH2-catalyzed NO formation accounts for only about 5% of total GTN turnover [16]. Thus, low rates of denitration could be accompanied by sufficiently high rates of bioactivation in an efficient pathway of GTN denitration that yields stoichiometric amounts of NO or a related sGC activator.

Activation of endothelial NO synthase by GTN itself was considered as alternative explanation for GTN bioactivity [31]. However, the non-selective NO synthase inhibitor l-NNA did not antagonize but slightly potentiated the effect of GTN, excluding the involvement of endogenous NO synthesis. The observed leftward shift of the response to DEA/NO and GTN in the presence of l-NNA was relatively small and not further investigated. The short time frame of the experiments excludes up-regulation of sGC expression, but it is conceivable that l-NNA blocked inactivation of NO by superoxide, which may be generated by uncoupled NO synthase in GTN-exposed blood vessels [39].

We speculated that ALDH2-independent GTN bioactivation in porcine and bovine coronary arteries might be identical to the low-affinity pathway mediating GTN vasodilation in ALDH2-deficient murine blood vessels. Comparison of GTN potency in vessels obtained from different species was hampered by a pronounced effect of precontraction levels. Lowering precontraction levels of rat aortic rings by about 50%, to mimic the levels applied to porcine and coronary arteries, led 5- to 10-fold potentiation of the effects of GTN and DEA/NO (cf. Fig. 2). Therefore, we calculated GTN potency relative to the potency of DEA/NO. The ratios of the respective EC_50_ values suggest that the ALDH2-independent porcine and bovine pathways exhibit about 5-fold lower potency than the ALDH2-catalyzed reaction in rodents. Published data with ALDH2 knockout mice, however, point to a more than 100-fold difference in potency of the high and low affinity pathways (EC_50_ = 0.1 and 12 μM, respectively; [15]), indicating that the ALDH2-independent reaction described here is not the same that is involved in the low affinity effects of GTN in rodents.

Thus, GTN appears to be bioactivated in porcine and bovine blood vessels through an unknown reaction not involving ALDH2. Obviously, it would be interesting to identify the responsible enzyme. Based on a recent report [34], we considered ALDH3A1 as potential candidate. Although chloral hydrate is generally believed to be a non-selective ALDH inhibitor, we found no conclusive evidence showing that this drug inhibits ALDH3A1. Therefore, we tested the selective ALDH3A1 inhibitor CB25 [28], but observed no effect on GTN-induced relaxation of rat aorta or porcine and bovine coronary arteries. These results, which agree well with the lack of significant ALDH3A1 mRNA expression levels (cf. Fig. 5) in these blood vessels, appear to exclude a significant contribution of ALDH3A1 to vascular GTN bioactivation.

In addition, several compounds interfering with bioactivation pathways proposed previously, in particular cytochrome P450 and GSH-transferase, had no considerable effects or were unsuitable for various reasons. Thus, we attempted to characterize this pathway biochemically and measured GTN-induced cGMP accumulation in homogenates and subcellular fractions of porcine coronary arteries with and without exogenously added sGC purified from bovine lung. However, GTN sensitivity was almost completely lost upon homogenization of the tissue, partly due to SOD- and DPI-insensitive scavenging of NO (Kollau, A., Neubauer, A. Russwurm, M., Koesling, D. and Mayer, B.; unpublished results). Further work is going on in our laboratory to settle this issue.

Taken together, our results provide evidence for an efficient and potent ALDH2-independent pathway of GTN bioactivation in porcine and bovine coronary arteries. If present in human blood vessels, this pathway might contribute to the therapeutic effect of organic nitrates that are not metabolized by ALDH2.

## Figures and Tables

**Table 1:** PCR primers used for qPCR analysis.

Gene	Forward primer	Reverse primer	GeneBank accession no. (NCBI)	Efficiency
**Rat aorta**				
ALDH1A1	5′-ttaaccctgcaactgaggag-3′	5′-caaagactttcccaccattg-3′	NM_022407	1.99
ALDH2	5′-aacgtggtggtgatgaaagt-3′	5′-gtgaccaacctcagtggaac-3′	NM_032416	1.86
ALDH3A1	5′-catcatgtacactgggagca-3′	5′-tccacgatttggttctgaat-3′	NM_031972	1.87
Cyclophilin A	5′-ctggtggcaagtccatctac-3′	5′-cccgcaagtcaaagaaatta-3′	NM_017101	2.01

**Bovine coronary artery**				
ALDH1A1	5′-caaaccagcagagcaaaccc-3′	5′-gagaagaaatggctgcccct-3′	NM_174239	1.91
ALDH2	5′-aaccttccccacggtcaatc-3′	5′-ttcactgctctgtccacgtc-3′	NM_001075367	1.82
ALDH3A1	5′-gacccctctatccagagcca-3′	5′-gaagtgccgggagttgatga-3′	NM_001166513	2.77
Cyclophilin A	5′-aagactgagtggttggatg-3	5′-gtcagcaatggtgatcttc-3′	NM_178320	1.97

**Porcine coronary artery**				
ALDH2	5′-acaaggaagatgtggacaggg-3′	5′-tctccagtgccgctaggt-3′	NM_001044611	2.16
ALDH3A1	5′-agtcccgtgactacggaaga-3′	5′-ctccgtaggcgactttctgg-3′	XM_005669258	2.17
Cyclophilin A	5′-cgtcttcttcgacatcgccg-3′	5′-ctttctccccagtgctcaga-3′	NM_214353	1.91
